# Mitochondrial DNA 3243A>T mutation in a patient with MELAS syndrome

**DOI:** 10.1038/s41439-018-0026-6

**Published:** 2018-09-04

**Authors:** Takahiro Ikeda, Hitoshi Osaka, Hiroko Shimbo, Makiko Tajika, Masayo Yamazaki, Ayako Ueda, Kei Murayama, Takanori Yamagata

**Affiliations:** 1 Division of Pediatrics, Jichi Medical University, Shimotsuke, Tochigi Japan; 2 Department of Pediatric Neurology, Kanagawa Children’s Medical Center, Yokohama, Kanagawa Japan; 3 Department of Metabolism, Chiba Children’s Hospital, Chiba, Chiba, Japan

## Abstract

Approximately 80% of cases of mitochondrial myopathy, encephalopathy, lactic acidosis, and stroke-like episodes (MELAS) harbor a heteroplasmic m.3243A>G transition in the tRNA^Leu (UUR)^ (*MTTL1*) gene. We report a MELAS case with a rare heteroplasmic m.3243A>T mutation found by direct sequencing of *MTTL1*. This mutation has been previously reported in 5 cases, of which 2 cases had the MELAS phenotype. Our case also strengthens the hypothesis that the m.3243A>T mutation can cause the MELAS phenotype.

Mitochondrial myopathy, encephalopathy, lactic acidosis, and stroke-like episodes (MELAS) syndrome is caused by mitochondrial DNA (mtDNA) abnormalities^1,2^. More than 80% of MELAS cases have a common m.3243A>G mutation in the mitochondrial tRNA^Leu (UUR)^ (*MTTL1*) gene^3,4^. PCR followed by *Apa*I digestion, which detects the A to G substitution at position 3243, is commonly used as a screening tool^4^. However, this approach cannot detect an A to T mutation at the same position, which is another nucleotide change that does not generate the *Apa*I site. We identified an m.3243A>T mutation in a patient with MELAS and compared the phenotypes associated with the rare A>T and the common A>G mutations at m.3243.

The patient is a 15-year-old boy from nonconsanguineous parents. His older sister suffered from depression, and his grandmother had diabetes mellitus. He showed normal development and growth until 11 years of age, when he presented with generalized tonic-clonic seizures. At the age of 13 years, he experienced partial seizures that started from the left side of the face and extended to generalized tonic-clonic seizures that occurred several times a year. Carbamazepine, lamotrigine, and topiramate were administered for seizure control. His academic performance declined, and his intelligence quotient was assessed as 67 with WISC-III before he was admitted to our hospital at 14 years of age. In that same period, he lost 10% of body weight in a 3-month period. On admission, his height and weight (157 cm, −0.6 SD; 45 kg, −0.6 SD, respectively) were normal. He showed cerebellar symptoms, such as dysmetria and intention tremor. He also presented with mild muscle weakness (MMT: 4/5) at the left upper and lower extremities. Deep tendon reflexes were symmetrical and within normal limits. Pathological reflexes were not observed. Despite normal results of routine blood investigations, including lactate and pyruvic acid (13.9 and 0.63 mg/dL; normal range 3.7–16.3 and 0.30–0.90 mg/dL, respectively), cerebrospinal fluid (CSF) lactate and pyruvic acid were elevated (35.7 and 1.37 mg/dL; normal range 13.7–20.5 and 0.63–0.77 mg/dL, respectively). Fasting blood glucose was 93 mg/dL, and urinary sugar was negative. Brain magnetic resonance imaging (MRI) demonstrated high-intensity areas of small multifocal gray matter regions in the right temporal, parietal, and occipital lobes, and diffuse white matter lesions in the left temporal lobe on T2 and fluid-attenuated inversion recovery (FLAIR) images (Fig. 1a; left). No regions followed vascular territories or demonstrated restricted diffusion, thus indicating a subacute/chronic pattern. Auditory brain responses revealed no abnormalities.

**Fig. 1:**
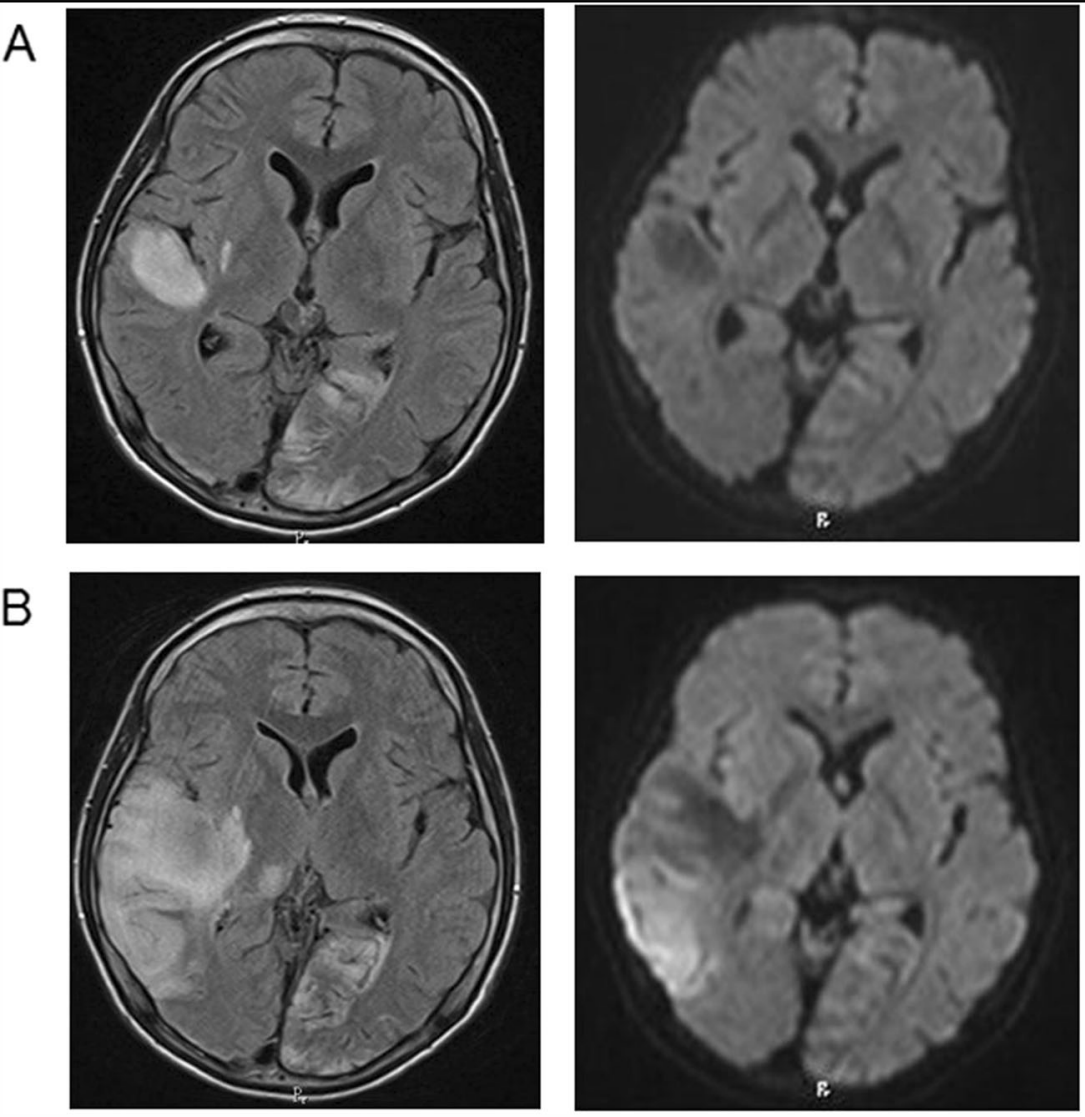
Sequential changes in MRI. (**a**) Brain MRI on admission shows small multifocal gray matter regions in the right temporal, parietal, and occipital lobes, and diffuse white matter lesions in the left temporal lobe, visualized as high-intensity areas in FLAIR images (left). White matter lesions in the left temporal lobe show low intensity in diffusion-weighted images (right). **b** Extended white matter and left thalamic lesions appeared after a stroke-like episode

After admission, he suffered a stroke-like episode that consisted of frequent generalized tonic-clonic seizures, prolonged consciousness disturbance, and left hemiplegia with expansion of the MRI lesions (Fig. 1b). Intravenous injections of arginine for 3 days, mannitol for 10 days, and edaravone for 14 days were used. Oral arginine, coenzyme Q10, vitamin B1 and B6 were also initiated. His left hemiplegia and mild cerebellar dysfunction remained.

Stroke-like episodes, CSF, and brain MRI findings were consistent with MELAS syndrome. After obtaining informed consent, his DNA was extracted from white blood cells. PCR for *MTTL1* and *Apa*I digestion did not identify a new restriction site introduced by the A to G substitution at position 3243. Direct sequencing of *MTTL1* and mitochondrially encoded NADH dehydrogenase 5 (*ND5*) from blood was performed, and an m.3243A>T mutation was found in *MTTL1*. This mutation created a new restriction site for the *TspR*I enzyme. PCR was performed using a forward primer (m.3114–3134) and a reverse primer (m.3432–3451). The amplified products were digested with *TspR*I at 65 °C for 1 h and separated in 2.5% agarose gels. Estimated levels of heteroplasmy were 22% in blood, 31% in nail, 37% in saliva, 26% in hair, and 35% in fibroblasts (Fig. 2, Suppl. Figure 1). RNA-folding analysis predicted different secondary structures when A, G, or T occupied position 3243 (Mfold; http://unafold.rna.albany.edu/?q=mfold/mfold-references; Suppl. Figure 2). We measured the oxygen consumption rate (OCR) in fibroblast by using an extracellular flux analyzer (Seahorse XF96; Agilent, Santa Clara, CA, USA). Compared to the maximum respiration rates in the control, the maximum respiration rates were reduced to 58% in glucose medium and 47% in galactose medium. The activities of the respiratory chain complexes were within normal range.

**Fig. 2:**
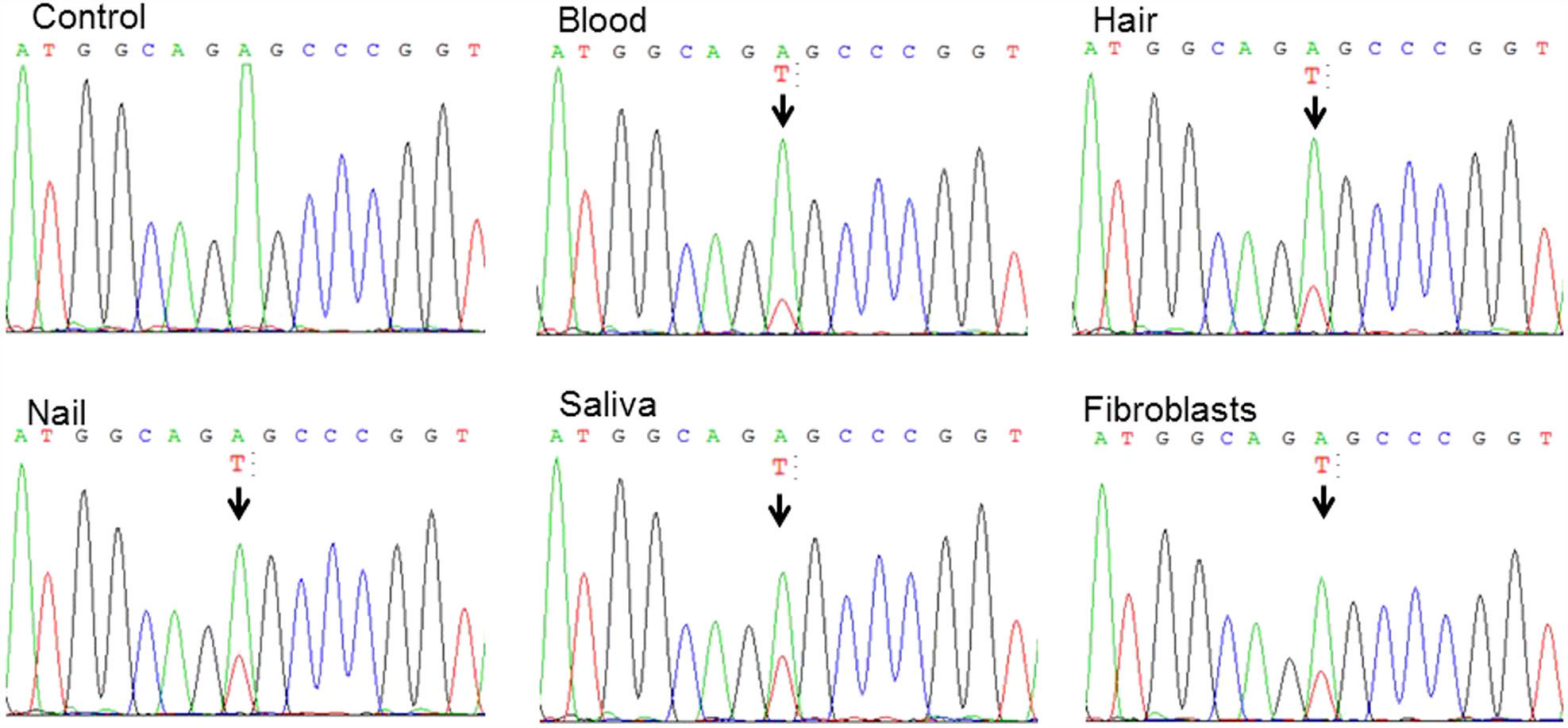
Direct sequencing of *MTTL1* from blood revealed the m.3243A>T mutation. Sequencing chromatogram of the *MTTL1* gene shows the heteroplasmic m.3243A>T mutation in the control sample and in blood, hair, nail, saliva, and fibroblasts from the patient

Although magnetic resonance spectroscopy and muscle biopsy were not performed, a stroke-like episode, MRI findings, and elevation of lactate in CSF suggested the diagnosis of MELAS syndrome in this case. Therefore, PCR of *MTTL1* and digestion with *Apa*I to detect a new restriction recognition site generated by m.3243A>G, which is the major cause of MELAS, were performed^4,5^. However, this approach was unable to detect the G to T substitution that was present in this case. Then, we performed direct sequencing of *MTTL1* and identified the m.3243A>T mutation. We also detected a decrease in the maximum respiration rate of OCR. To the best of our knowledge, the m.3243A>T mutation has been reported in 5 cases (Table 1). Shaag et al. reported a case of a 9-year-old female with rapid progressive encephalopathy and marked lactic acidosis^5^. She presented with a loss of appetite, progressive muscle weakness, and exercise intolerance before the onset of encephalopathy. She then moved into a vegetative state at 10 years of age. Longo et al. also reported a case of a girl with a severe MELAS phenotype with recurrent cerebral infarcts who died at 9 years of age^6^. However, Alston et al. reported two cases that did not have MELAS syndrome: one adult female with normal intellect, sensorineural deafness, and short stature; the other was also a female with chronic progressive external ophthalmoplegia^7^. Czwell et al. also reported an adult case of exercise-induced myalgia and rhabdomyolysis. The patient was normal, except for muscle ache after physical exercise^8^. Including our case, 3 of the 6 cases with the m.3243A>T mutation presented with the MELAS phenotype. Although the secondary structure is predicted to be different between the G and T mutants at m.3243 (Suppl. Figure 2), there are no differences in clinical features between the 3 observed cases with the m.3243A>T mutation and those with the m.3243A>G mutations (Table 1).

**Table 1:** Cases with m.3243A>T mutation

Reference	Case	Sex	Onset	Phenotype	Family history	Presentation	Images	Prognosis	Mutation rate	
Shaag et al. ^5^	1	F	6 y	MELAS	N.A.	Encephalopathy Muscle weakness Fatigue	CT: ventriculomegaly	10 y vegetative state	Muscle skin blood	81% 69% 14%
Longo et al.^6^	2	F	7 y	MELAS	N.A.	Stroke-like episodes Hearing loss Visual impairment Short stature Fatigue	MRI, MRA: partial atrophy, multi embolisms	9 y died	Muscle blood skin	50% 50% 50%
Alston et al.^7^	3	M	6 y	Hearing loss	None	Hearing loss Recurrent keratitis Short stature	CT: basal ganglia calcification	22 y normal IQ	Muscle urinary blood buccal	87% 88% 46% 16%
	4	F	8 y	CPEO	Mother and aunt had short stature	Visual impairment Muscle weakness Ophthalmoplegia Short stature	N.A.	N.A.	Blood	5–10%
Czell et al.^8^	5	M	29 y	Rhabdomy-olysis	Two cousins had diabetes mellitus	Muscle ache after physical exercise	N.A.	N.A.	Muscle	30%
Our case	6	M	11 y	MELAS	Grandmother had diabetes mellitus Sister had depression	Stroke-like episodes Visual impairment Muscle weakness Short stature	MRI: multi-focal stroke like lesions		Blood nail saliva hair fibroblasts	22% 31% 37% 26% 35%

*urinary* urinary sediment, *buccal* buccal epitheria, *MELAS* mitochondrial myopathy, encephalopathy, lactic acidosis, and stroke-like episodes, *CPEO* chronic progressive external ophthalmoplegia, *N.A*. not available

In addition to m.3243A>G, mutations causing MELAS mainly include m.3271T>C (~7.5%) and m.3252A>G (<5%) in *MTTL1* and m.13513G>A (<15%) in *ND5*^4^. As the screening m.3243 with *Apa*I digestion is routinely performed, the m.3243A>T transition may have been missed in other cases. Accumulation of new cases with the m.3243A>T mutation may reveal the clinical characteristics of this mutation.

## Electronic supplementary material

Supplementary figure 1

Supplementary figure 2

## Data Availability

The relevant data from this Data Report are hosted at the Human Genome Variation Database at 10.6084/m9.figshare.hgv.2369
