# Supplementary figures and images for: Mitochondrial DNA 3243A>T mutation in a patient with MELAS syndrome
Source: Hum Genome Var. 2018 Sep 4;5:25. doi: 10.1038/s41439-018-0026-6 (PMC6123423; doi:10.1038/s41439-018-0026-6)

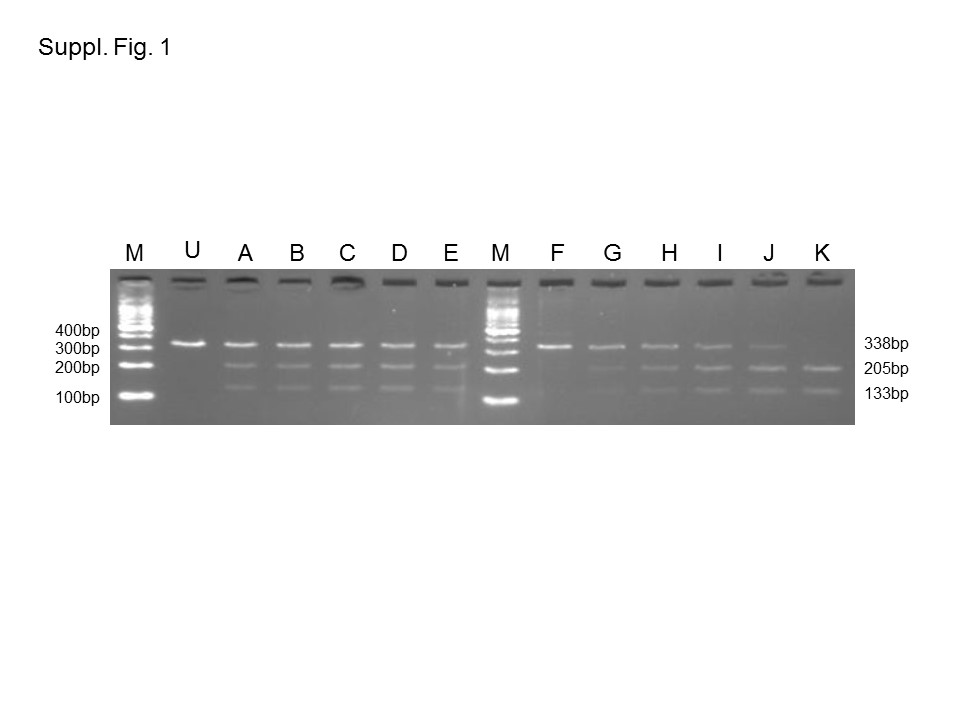

Supplement: Supplementary file 1 — Supplementary figure 1 [file 41439_2018_26_MOESM1_ESM.jpg]

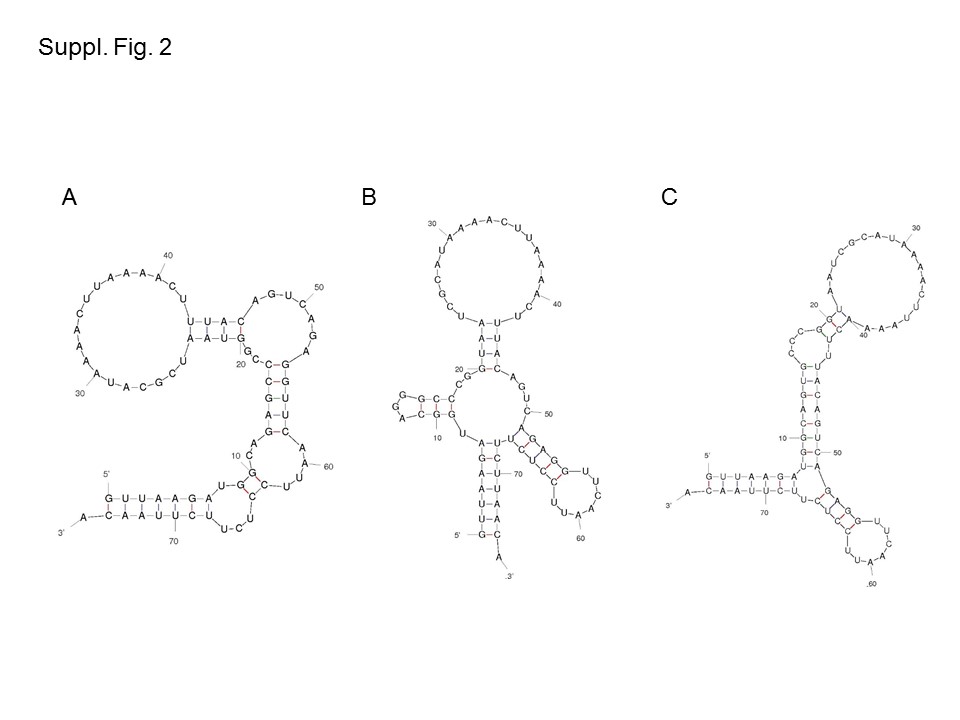

Supplement: Supplementary file 2 — Supplementary figure 2 [file 41439_2018_26_MOESM2_ESM.jpg]
